# The use of the endoscope in endodontic surgery: A systematic review

**DOI:** 10.4317/jced.56539

**Published:** 2020-10-01

**Authors:** Antonio Pallarés-Serrano, Pablo Glera-Suarez, David Soto-Peñaloza, David Peñarrocha-Oltra, Thomas von Arx, Miguel Peñarrocha-Diago

**Affiliations:** 1 DDS, MS. Master in Oral Surgery and Implant Dentistry, Department of Stomatolo-gy, Faculty of Medicine and Dentistry, University of Valencia, Spain; 2 DDS. Master in Oral Surgery and Implant Dentistry, Department of Stomatology, Faculty of Medicine and Dentistry, University of Valencia, Spain; 3 DDS, PhD. Assistant Professor, Oral Surgery Unit, Department of Stomatology, Faculty of Medicine and Dentistry, University of Valencia, Spain; 4 Prof. Dr. med. dent. Department of Oral Surgery and Stomatology, School of Dental Medicine, University of Bern, Bern, Switzerland; 5 MD, PhD, DDS. Full Professor, Oral Surgery Unit, Department of Stomatology, Faculty of Medicine and Dentistry, University of Valencia, Spain

## Abstract

**Background:**

A systematic review of clinical studies with at least one year of follow-up was done to assess the success rate of endodontic surgery including endoscopy for magnification and illumination.

**Material and Methods:**

Five electronic databases were searched, including MEDLINE (via PubMed), Embase, Web of Science, Scopus and the Cochrane Library of the Cochrane Collabora-tion (CENTRAL). There were no language restrictions, and the search covered the period up to October 2019. The risk of bias was evaluated with the Cochrane Collaboration tool for randomized clinical trials and the ROBINS-I tool for non-randomized studies of inter-ventions.

**Results:**

From the 278 initially identified titles, finally 2 randomized controlled trials and 3 non-randomized studies met the inclusion criteria. All the included studies analyzed the success rate of endodontic surgery performed with the help of endoscope for magnifica-tion and illumination. The risk of bias was high for allocation sequence concealment and blinding of participants and personnel in the randomized controlled trials. The nonran-domized studies showed limitations in terms of confounding bias and blinding of outcome assessment. Endodontic surgery with the help of an endoscope is associated with high success rates (88.9-94.9%).

**Conclusions:**

The endoscope was associated with high success rates of endodontic sur-gery in the included studies. Future studies on this topic are warranted, due to the meth-odological issues and the scarce number of randomized clinical trials.

** Key words:**Endodontic surgery, magnification, endoscope, success.

## Introduction

Microsugery is defined as a surgical procedure on exceptionally small and complex structures. Microsurgery starts with a magnification of at least eight. That can be achieved with a dental operating microscope or endoscope (1).

The use of endoscope has brought many advantages to endodontic surgery, including a smaller ostectomy size or the reduction of the resection angle at the tip of the root to reduce the number of exposed tubules in the apical zone (2,3).

Higher magnification and illumination are also very favorable for examining the resected root surface while methylen blue dye is used for both, staining the periodontal ligament to ensure complete resection of the root, and looking for cracks, isthmuses and extra canals (4).

The introduction of microsurgical instruments and ultrasonic tips has been shown to improve the outcomes of endodontic surgery in comparison with the traditional technique (5-7). Preparation of the retrograde cavities is easier, safer and more precise than when conventional handpieces are used (5).

Although microscopes and loupes provide the illumination needed for endodontic surgery, adherence to a strict endodontic surgical protocol is suggested to attain high success rates (5). There has been growing interest in endoscopy as an alternative method for adequate illumination and magnification in endodontic surgery, since it provides correct visibility and is easier to use than other devices (2,6,7).

The endoscope could also be used to check the quality of root-end filling and de-tect dentinal cracks after root-end resection (8-10).

Thus, the present systematic review of clinical studies involving a follow-up period of at least one year was carried out to assess the success rate of endodontic surgery performed with the help of endoscopy for magnification and illumination.

## Material and Methods

The systematic review was carried out based on the principles of the Transparent Reporting of Systematic Reviews and Meta-Analyses - PRISMA Statement (11) and AM-STAR-2 guidelines (12).

-Focus question

The research question was established based on the adaptation of structured PICO question, in this case applying a PIO (population, intervention, outcome). The question format was as follows.

“What is the success rate of endodontic surgery including an endoscope?”

P (population): Patients requiring endodontic surgery.

I (intervention): Endodontic surgery using an endoscope for magnification and illumination.

O (outcome): Success rate after follow-up of at least one year.

-Search strategy

Five electronic databases were searched: MEDLINE (via PubMed), Embase, Web of Science, Scopus and the Cochrane Library of the Cochrane Collaboration (CENTRAL). There were no language restrictions, and the search covered the period up to October 2019. The search strategy included a combination of the controlled terms (MeSH and EMTREE), and key words were used whenever possible in order to secure the best search results. In addition, a manual search was conducted of the main topics related to the principal question, and the reference lists of the finally included articles were consulted to find other potentially eligible studies according to Greenhalgh and Peacock (13). Further-more, the Really Simple Syndication (RSS) feed resource from PubMed, was used to detect potential eligible titles fitting to the search strategy.

The following search strategy was used in Medline via Pubmed: (“Apicoectomy”(Mesh) OR periapical surgery OR apicoectomy OR apicectomy OR periradicular surgery OR root-end surgery OR root-end filling OR endodontic surgery OR surgical endodontic treatment OR endodontic microsurgery OR retro-grade filling OR retro-grade surgery OR apical microsurgery) AND (“Endoscopes”(Mesh) OR endoscope OR endoscopy OR endoscopic OR dental endoscope) AND (outcome OR treatment outcome OR healing OR success OR success ratio OR complications). The search strategies fitted to different data-bases are provided in the supplementary material (Appendix-S1).

-Inclusion and exclusion criteria

The articles were included in the systematic review if they complied with the following criteria: randomized clinical trial (RCT), non-randomized study (NRS) in humans, assessing the impact of endoscopy used for magnification and illumination upon the success of endodontic surgery, with a minimum follow-up period of 12 months, and providing information related to the success of the surgical procedure. Case reports, literature reviews, expert opinions, *in vitro* or nonclinical studies were excluded from the systematic review.

-Screening and selection of papers

Two independent reviewers (APS, PGS) performed the literature and manual searches, the same reviewers extracted the data from the publications (APS, PGS). Studies failing to meet the inclusion criteria were excluded. In the event of disagreement, consensus was reached through discussion with a third reviewer (MPD).

-Risk of bias in individual studies

Two independent reviewers (APS and PGS) evaluated all included articles. The methodological quality of the studies was evaluated using the Cochrane Collaboration tool to as-sess the risk of bias for randomized clinical trials, while the ROBINS-I (Risk of Bias In Non-randomized Studies of Interventions) tool was used for non-randomized studies (14). For each aspect of the quality assessment, the risk of error was based on the recommendations of the Cochrane Handbook for Systematic Reviews of Interventions 5.1.0 (http://handbook.cochrane.org). The criteria for each entry were: “yes” (= low risk of bias), “no” (= high risk of bias) or “uncertain” (due to lack of information or uncertainty regarding the potential of bias). A study was considered to have “low” risk of bias in the presence of an adequate sequence of generation, allocation concealment and blinding (patients and personnel). If one or more criteria were not met, the study was considered to have “high” risk of bias. Disagreements between reviewers were solved by discussion with a third advisor (DSP). The level of reviewer’s agreement was assessed by Kappa values, and interpreted using the Landis and Koch scale (15).

-Summary of measures and synthesis of results 

The data were entered in an Excel spreadsheet (Microsoft Corporation, Redmond, WA, USA). The following information was compiled, seeking comparability between studies: authors and year, sample size, magnification approach, follow-up in months, root-end preparation instruments, root-end filling material, success rate and study design.

–Definition of success criteria: The included studies assessed the healing according to the criteria by Molven *et al.* (16,17). Complete healing, incomplete healing, uncertain healing, or unsatisfactory outcome. Signs and symptoms were also recorded and classified as clinical success, clinically questionable, and clinical failure.

## Results

-Study selection

**Figure 1:**
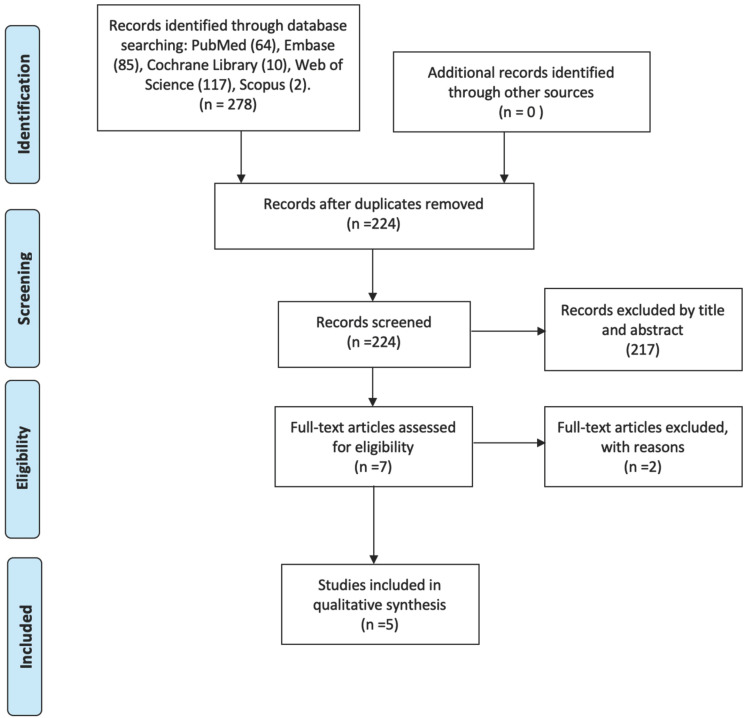
PRISMA flowchart of the selection of studies.

A total of 278 articles were found by the initial search. After duplicates removal, 224 papers were screened by title and abstract and 7 were identified as potentially eligible for inclusion. These 7 articles underwent full-text assessment. From these studies, 2 studies were excluded (18,19) (Table 1) and 5 articles were found to meet the inclusion criteria and were finally included in the systematic review (Fig. 1).

**Table 1:**
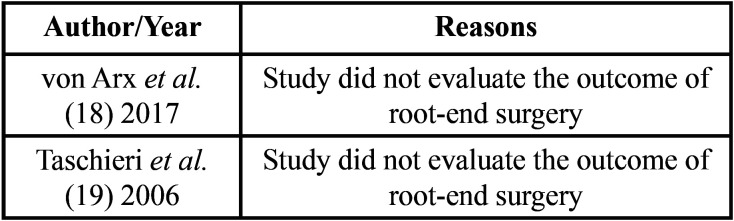
Reasons for studies to be excluded from this systematic review, with reasons.

-Study characteristics

We finally included two randomized clinical trials (RCTs) and three prospective case series (2,5-7,20). No retrospective clinical studies were identified. The retrograde obturation material included zinc-eugenol EBA-reinforced cement (SuperEBA) in all included studies. The RCT published by Taschieri *et al.* compared endoscopy versus a control group in which surgical loupes were used (5). In contrast, the RCT by Taschieri *et al.* used light microscopy for endodontic surgery in the control group (6), and the prospective trial published by von Arx *et al.* used no magnifying device in the control group (20). In the study carried out by von Arx *et al.*, the success rate in the endos-copy group was 88.9% versus only 75.4% in the control group (no magnification device). Nevertheless, the differences between the groups failed to reach statistical significance (20).

The RCT published by Taschieri *et al.* (compared endodontic surgery with an endoscope versus a control group in which loupes were used. Although the differences between the groups failed to reach statistical significance, the success rate was higher in the endoscopy group (94.9%) than in the control group (90.6%). The authors reported that surgery took longer to be completed with the endoscope than with the loupes (5).

In another RCT, Taschieri *et al.* compared endoscopy versus a control group in which a microscope was used for magnification and illumination. Both endoscopy and microscopy yielded high success rates (90% and 92%, respectively) (6). The characteristics of the five studies included in the systematic review are described in Table 2.

**Table 2:**
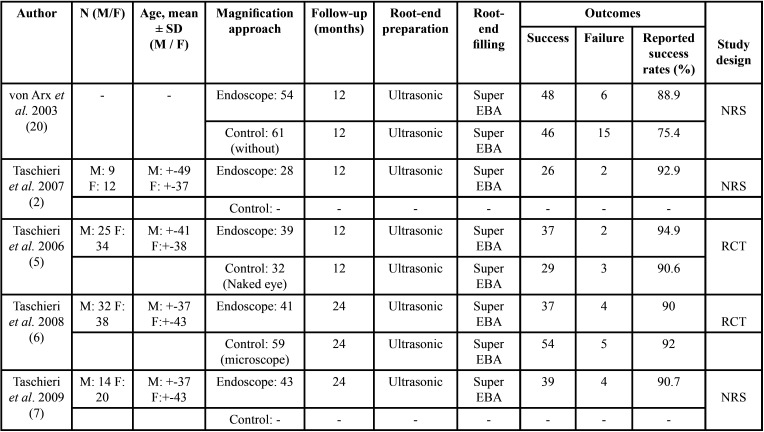
Descriptive summary of the studies included in this systematic review.

-Risk of bias across studies

**Figure 2:**
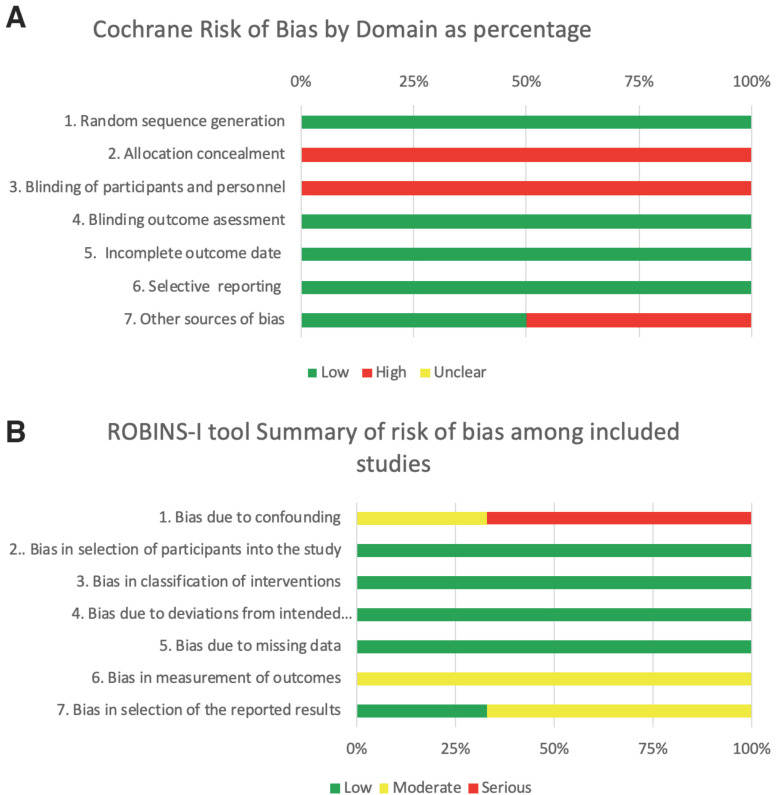
Summary of risk of bias as percentage per variable. (A) Randomized controlled trials. (B) Non-randomized studies.

Allocation concealment was not clearly described in RCTs. The second least reported item was blinding of the patients (Fig. 2). The methodological quality of the three nonrandomized studies was evaluated using the ROBINS-I tool (14), which found all three to have moderate risk of bias. The inter-reviewer agreement was almost perfect (k=0,87). The judgement reasons during methodological appraisal is provided in descriptive manner in the supplementary material (Appendix S4-S5).

## Discussion

The main objective of this systematic review was to assess the success rate of endodontic surgery performed with the help of endoscopy for magnification and illumination, over a follow-up period of at least one year.

We included two RCTs with high risk of bias related to allocation concealment and the blinding of participants, nevertheless, there is evidence that surgical studies are difficult to blind, and three NRS with high risk of confounding bias, since the statistical analysis in these studies failed to control for relevant confounders (e.g. patient age and gender). The use of an endoscope was associated with a high success rate in endodontic surgery (88.9-94.9%). In the endodontic literature, *in vitro* studies showed that magnification devices, such as the microscope or the endoscope, allow the identification of microstructures not visible with the naked eye (10,21,22). An experimental study by von Arx *et al.* reported that the endoscope accurately identified microstructures following root-end resection and root-end preparation. The endoscope could be considered for use during intraoperative diagnostics in endodontic surgery (22). The same author demonstrated in a clinical study about endodontic surgery with an intraoperative endoscopic examination of first permanent molars a high frequency of canal isthmuses at the apex resection level. Endoscopic inspection also demonstrated that none of the isthmuses were filled, emphasizing the difficulty of orthograde instrumentation and root filling of canal isthmuses (23). This latter aspect is of utmost importance, because of the limited field of view and difficult handling of the endodontic microscope in posterior zones.

von Arx *et al.* compared the microscopy and endoscopy with scanning electron microscopy, and the endoscopy x64 proved the most accurate visual aid for the identification of dentinal cracks after root-end resection in extracted human teeth; however, it also provided the most false identifications (10). Setzer *et al.* published a meta-analysis in 2011 comparing the surgical technique of endodontic surgery with the use of higher magnification devices (microscope or endoscope) and the technique without magnification or using only loupes. Although the study did not differentiate between microscope or endoscope, they reported that the probability of success with higher magnification devices proved to be significantly greater (24). Although the studies using endoscopy for endodontic surgery reported high success rates, further randomized controlled trials are needed in order to confirm these findings.

Note to mention, that success rates in endodontic procedures not only laid on the augmentation devices used, but also on the patient characteristics (e.g. systemic condition, gender, age), tooth features and position (e.g. anterior zone, multirooted tooth) (25). Thus, an ac-curate diagnosis is needed for a favourable prognosis and hinges to a careful patient selection (25,26).

Regarding the advantages of this approach, the field depth perception of the operator using an endoscope is quite similar to that with the naked eye – a fact that facilitates the use of the device (2,7). On the other hand, the field depth cannot be adjusted to the objective from different angles, in most cases a mirror must be used for indirect vision, while the endoscope allows easy adjustment, with observation from different angles, and provides direct images (7).

A report from von Arx *et al.* described better surgical outcomes (though without reaching statistical significance) with the endoscope than when micromirrors without magnifying devices were used (20). The endoscope also allows us to see behind the roots in an environment with good illumination. In comparison, movements of patient’s head are a limiting factor when using a microscope (6). The endoscope is versatile, rapid and comfortable to use (6). Adjustment to focus and zoom can be done with a single hand and is both rapid and convenient (7). In the case of the microscope, any movement of the latter or of the patient will cause the surgical field to move off-center when the magnification is increased (6). Nevertheless, the use of rigid endoscope should be a complementary device, and should not replace the microscope in endodontic microsurgery.

-Limitations of the present review

A limitation of our systematic review is the relative lack of studies comparing endodontic surgery assisted by endoscopy versus endodontic surgery without magnification or using other magnification devices. With regard to the methodological limitations, shortcomings were observed related to confounding bias and blinding of outcome assessment in the NRS design. In the case of the RCTs, allocation concealment and the blinding of participants and personnel showed high risk of bias. However, there is evidence that surgical studies are difficult to blind.

- Recommendations for future investigations

A number of recommendations can be made based on the findings of this systematic review. Future studies should seek to compare endodontic surgery assisted by endoscopy versus groups in which other magnifying devices are used, such as microscopes and loupes, with the objective of assessing variations among the different magnification and illumination strategies. Furthermore, a sufficiently large sample size is required, with follow-up periods long enough to allow the drawing of clear conclusions. It would also be interesting to design controlled studies involving endodontic surgery in which the microscope is compared with a group where both the microscope and endoscope are jointly used, as well as endodontic surgery in which surgical loupes are compared with a group where both surgical loupes and the endoscope are jointly used, but emending the methodological issues detected in the body of evidence on this topic.

## Conclusions

In conclusion, the use of an endoscope allows correct magnification and illumination to perform endodontic surgery and is associated with high success rates. Future studies on this topic are warranted, due to the methodological issues and the scarce number of randomized clinical trials.

## References

[1] Blahuta R, Stanko P (2012). The use of optical magnifying devices in periradicular microsurgery. Bratisl Lek Listy.

[2] Taschieri S, Del Fabbro M, Testori T, Weinstein R (2007). Endoscopic periradicular surgery: a prospective clinical study. Br J Oral Maxillofac Surg.

[3] von Arx T, Walker WA (2000). Microsurgical instruments for root-end cavity prepara-tion following apicoectomy: a literature review. Endod Dent Traumatol.

[4] Kratchman SI (2007). Endodontic microsurgery. Compend Contin Educ Dent.

[5] Taschieri S, Del Fabbro M, Testori T, Francetti L, Weinstein R (2006). Endodontic sur-gery using 2 different magnification devices: preliminary results of a randomized controlled study. J Oral Maxillofac Surg.

[6] Taschieri S, Del Fabbro M, Testori T, Weinstein R (2008). Microscope versus endoscope in root-end management: a randomized controlled study. Int J Oral Maxillofac Surg.

[7] Taschieri S, Del Fabbro M (2009). Endoscopic endodontic microsurgery: 2-year evalua-tion of healing and functionality. Braz Oral Res.

[8] Peñarrocha-Oltra D, Menéndez-Nieto I, Cervera-Ballester J, Maestre-Ferrín L, Peñarrocha-Diago M, Peñarrocha-Diago M (2019). Aluminum Chloride versus Electro-cauterization in Periapical Surgery: A Randomized Controlled Trial. J Endod.

[9] Peñarrocha-Diago M, Menéndez-Nieto I, Cervera-Ballester J, Maestre-Ferrín L, Blaya-Tárraga JA, Peñarrocha-Oltra D (2018). Influence of Hemostatic Agents in the Prognosis of Periapical Surgery: A Randomized Study of Epinephrine versus Aluminum Chloride. J Endod.

[10] von Arx T, Kunz R, Schneider AC, Bürgin W, Lussi A (2010 Sep). Detection of dentinal cracks after root-end resection: an ex vivo study comparing microscopy and endoscopy with scanning electron microscopy. J Endod.

[11] Moher D, Liberati A, Tetzlaff J, Altman DG; PRISMA Group (2009). Preferred report-ing items for systematic reviews and meta-analyses: the PRISMA statement. PLoS Med.

[12] Shea BJ, Reeves BC, Wells G, Thuku M, Hamel C, Moran J (2017). AMSTAR 2: a critical appraisal tool for systematic reviews that include randomised or non-randomised studies of healthcare interventions, or both. BMJ.

[13] Greenhalgh T, Peacock R (2005). Effectiveness and efficiency of search methods in sys-tematic reviews of complex evidence: audit of primary sources. BMJ.

[14] Sterne J, Hernán MA, Reeves BC, Savović J, Berkman ND, Viswanathan M (2016). ROBINS-I: a tool for assessing risk of bias in non-randomised studies of interventions. BMJ.

[15] Landis JR, Koch GG (1977). An application of hierarchical kappa-type statistics in the assessment of majority agreement among multiple observers. Biometrics.

[16] Molven O, Halse A, Grung B (1987). Observer strategy and the radiographic classifica-tion of healing after endodontic surgery. Int J Oral Maxillofac Surg.

[17] Molven O, Halse A, Grung B (1996). Incomplete healing (scar tissue) after periapical surgery--radiographic findings 8 to 12 years after treatment. J Endod.

[18] Von Arx T, Bosshardt D, Bingisser AC, Bornstein MM (2017). Endoscopic Evaluation of Cut Root Faces and Histologic Analysis of Removed Apices Following Root Resection: a Clinical Study. Eur Endod J.

[19] Taschieri S, Del Fabbro M, Testori T, Francetti L, Weinstein R (2006). Use of a surgical microscope and endoscope to maximize the success of periradicular surgery. Pract Proced Aesthet Dent.

[20] von Arx T, Frei C, Bornstein MM (2003). Periradicular surgery with and without en-doscopy: a prospective clinical comparative study. Schweiz Monatsschr Zahnmed.

[21] Slaton CC, Loushine RJ, Weller RN, Parker MH, Kimbrough WF, Pashley DH (2003). Identification of resected root-end dentinal cracks: a comparative study of visual magnification. J Endod.

[22] von Arx T, Montagne D, Zwinggi C, Lussi A (2003). Diagnostic accuracy of endoscopy in periradicular surgery - a comparison with scanning electron microscopy. Int Endod J.

[23] von Arx T (2005). Frequency and type of canal isthmuses in first molars detected by en-doscopic inspection during periradicular surgery. Int Endod J.

[24] Setzer FC, Kohli MR, Shah SB, Karabucak B, Kim S (2012). Outcome of endodontic surgery: a meta-analysis of the literature--Part 2: Comparison of endodontic mi-crosurgical techniques with and without the use of higher magnification. J Endod.

[25] Monaghan L, Jadun S, Darcey J (2019). Endodontic microsurgery. Part one: diagnosis, patient selection and prognoses. Br Dent J.

[26] Kim D, Lee H, Chung M, Kim S, Song M, Kim E (2020). Effects of fast- and slow-setting calcium silicate-based root-end filling materials on the outcome of endo-dontic microsurgery: a retrospective study up to 6 years. Clin Oral Investig.

